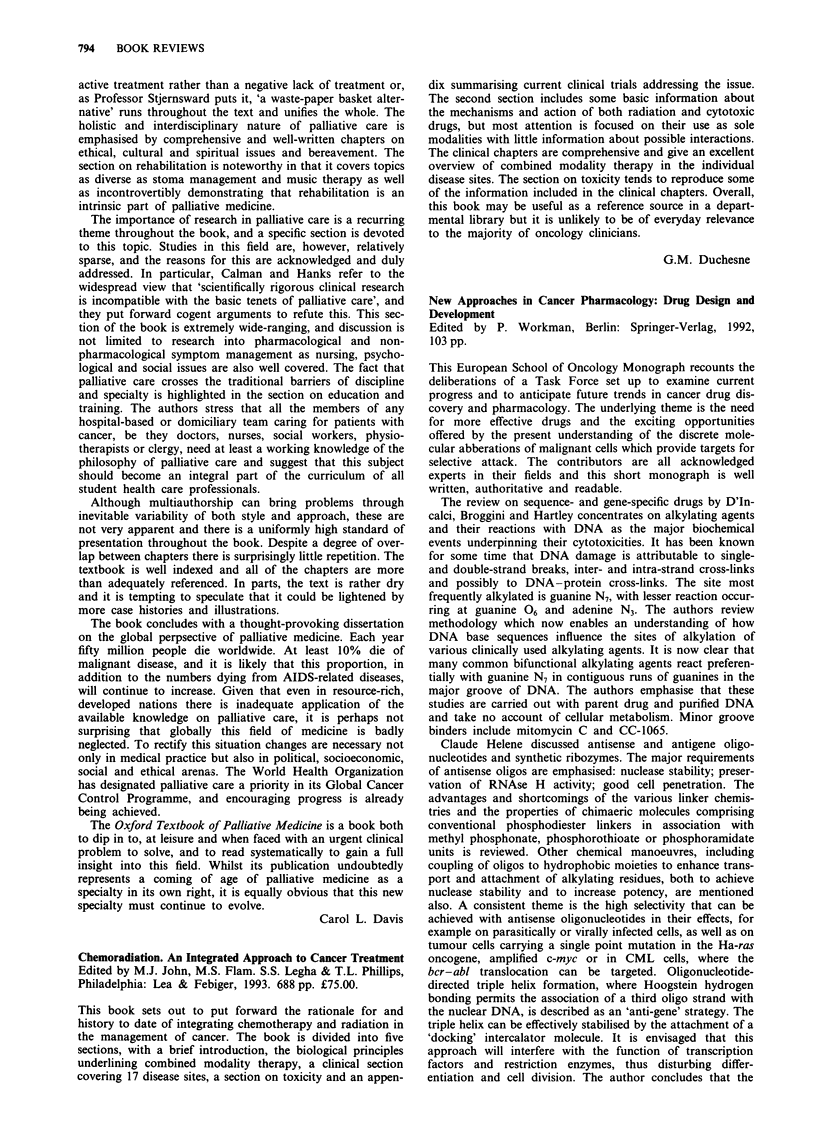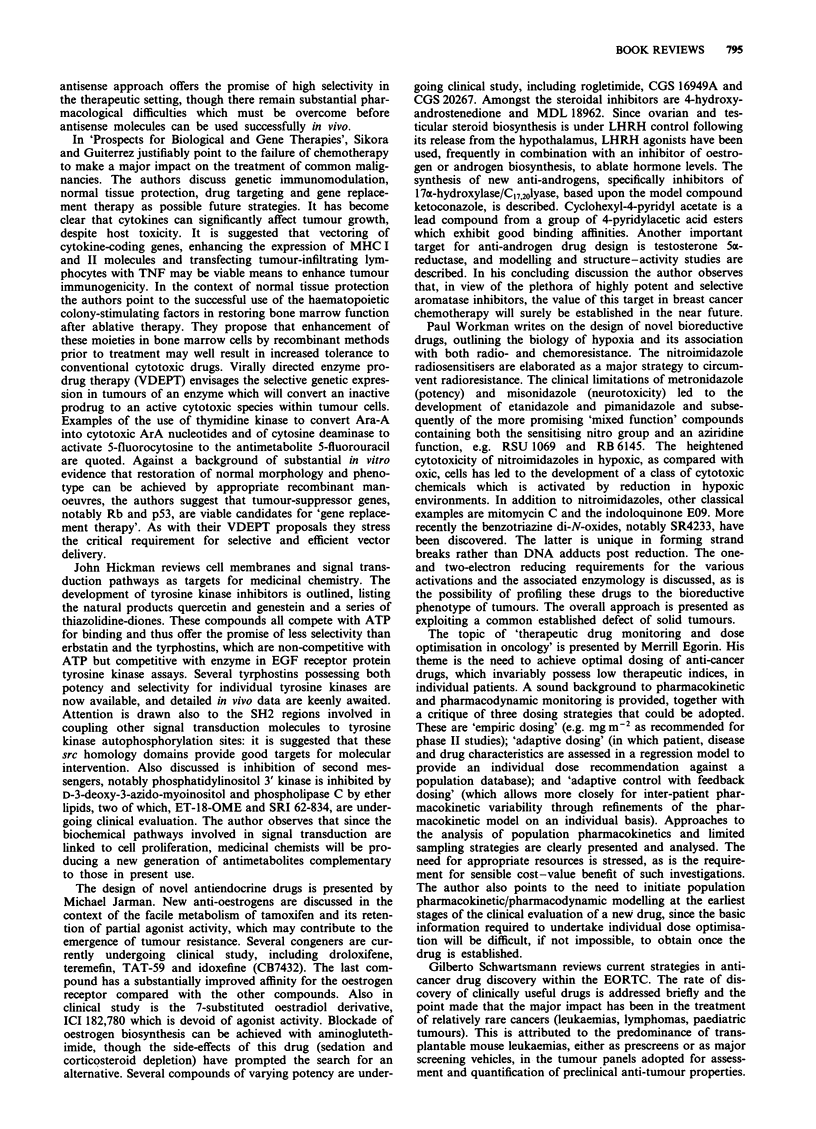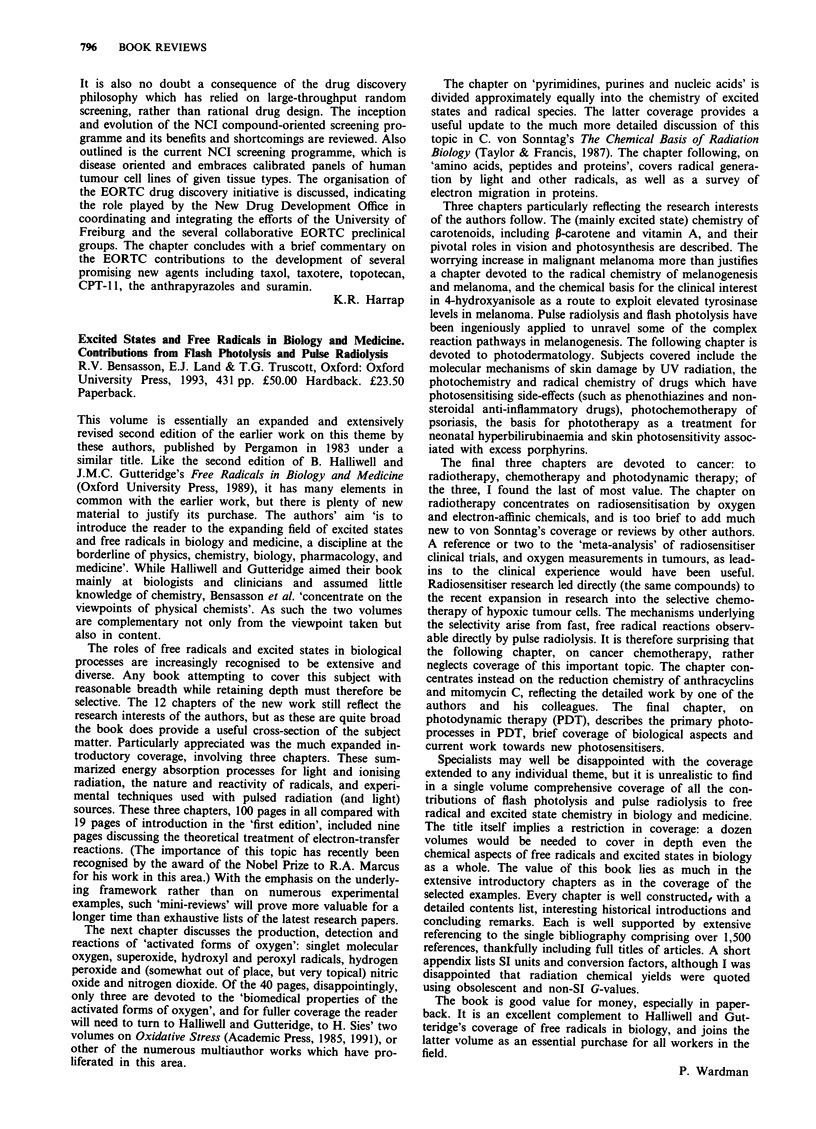# New Approaches in Cancer Pharmacology: Drug Design and Development

**Published:** 1994-04

**Authors:** K.R. Harrap


**New Approaches in Cancer Pharmacology: Drug Design and Development**
Edited by P. Workman, Berlin: Springer-Verlag, 1992, 103 pp.

This European School of Oncology Monograph recounts the deliberations of a Task Force set up to examine current progress and to anticipate future trends in cancer drug discovery and pharmacology. The underlying theme is the need for more effective drugs and the exciting opportunities offered by the present understanding of the discrete molecular abberations of malignant cells which provide targets for selective attack. The contributors are all acknowledged experts in their fields and this short monograph is well written, authoritative and readable.

The review on sequence- and gene-specific drugs by D'Incalci, Broggini and Hartley concentrates on alkylating agents and their reactions with DNA as the major biochemical events underpinning their cytotoxicities. It has been known for some time that DNA damage is attributable to single- and double-strand breaks, inter- and intra-strand cross-links and possibly to DNA–protein cross-links. The site most frequently alkylated is guanine $N_7$, with lesser reaction occurring at guanine $O_6$ and adenine $N_3$. The authors review methodology which now enables an understanding of how DNA base sequences influence the sites of alkylation of various clinically used alkylating agents. It is now clear that many common bifunctional alkylating agents react preferentially with guanine $N_7$ in contiguous runs of guanines in the major groove of DNA. The authors emphasise that these studies are carried out with parent drug and purified DNA and take no account of cellular metabolism. Minor groove binders include mitomycin C and CC-1065.

Claude Helene discussed antisense and antigene oligonucleotides and synthetic ribozymes. The major requirements of antisense oligos are emphasised: nuclease stability; preservation of RNAse H activity; good cell penetration. The advantages and shortcomings of the various linker chemistries and the properties of chimaeric molecules comprising conventional phosphodiester linkers in association with methyl phosphonate, phosphorothioate or phosphoramidate units is reviewed. Other chemical manoeuvres, including coupling of oligos to hydrophobic moieties to enhance transport and attachment of alkylating residues, both to achieve nuclease stability and to increase potency, are mentioned also. A consistent theme is the high selectivity that can be achieved with antisense oligonucleotides in their effects, for example on parasitically or virally infected cells, as well as on tumour cells carrying a single point mutation in the Ha-*ras* oncogene, amplified c-*myc* or in CML cells, where the *bcr–abl* translocation can be targeted. Oligonucleotide-directed triple helix formation, where Hoogstein hydrogen bonding permits the association of a third oligo strand with the nuclear DNA, is described as an 'anti-gene' strategy. The triple helix can be effectively stabilised by the attachment of a 'docking' intercalator molecule. It is envisaged that this approach will interfere with the function of transcription factors and restriction enzymes, thus disturbing differentiation and cell division. The author concludes that the antisense approach offers the promise of high selectivity in the therapeutic setting, though there remain substantial pharmacological difficulties which must be overcome before antisense molecules can be used successfully *in vivo*.

In 'Prospects for Biological and Gene Therapies', Sikora and Guiterrez justifiably point to the failure of chemotherapy to make a major impact on the treatment of common malignancies. The authors discuss genetic immunomodulation, normal tissue protection, drug targeting and gene replacement therapy as possible future strategies. It has become clear that cytokines can significantly affect tumour growth, despite host toxicity. It is suggested that vectoring of cytokine-coding genes, enhancing the expression of MHC I and II molecules and transfecting tumour-infiltrating lymphocytes with TNF may be viable means to enhance tumour immunogenicity. In the context of normal tissue protection the authors point to the successful use of the haematopoietic colony-stimulating factors in restoring bone marrow function after ablative therapy. They propose that enhancement of these moieties in bone marrow cells by recombinant methods prior to treatment may well result in increased tolerance to conventional cytotoxic drugs. Virally directed enzyme prodrug therapy (VDEPT) envisages the selective genetic expression in tumours of an enzyme which will convert an inactive prodrug to an active cytotoxic species within tumour cells. Examples of the use of thymidine kinase to convert Ara-A into cytotoxic ArA nucleotides and of cytosine deaminase to activate 5-fluorocytosine to the antimetabolite 5-fluorouracil are quoted. Against a background of substantial *in vitro* evidence that restoration of normal morphology and phenotype can be achieved by appropriate recombinant manoeuvres, the authors suggest that tumour-suppressor genes, notably Rb and p53, are viable candidates for 'gene replacement therapy'. As with their VDEPT proposals they stress the critical requirement for selective and efficient vector delivery.

John Hickman reviews cell membranes and signal transduction pathways as targets for medicinal chemistry. The development of tyrosine kinase inhibitors is outlined, listing the natural products quercetin and genestein and a series of thiazolidine-diones. These compounds all compete with ATP for binding and thus offer the promise of less selectivity than erbstatin and the tyrphostins, which are non-competitive with ATP but competitive with enzyme in EGF receptor protein tyrosine kinase assays. Several tyrphostins possessing both potency and selectivity for individual tyrosine kinases are now available, and detailed *in vivo* data are keenly awaited. Attention is drawn also to the SH2 regions involved in coupling other signal transduction molecules to tyrosine kinase autophosphorylation sites: it is suggested that these *src* homology domains provide good targets for molecular intervention. Also discussed is inhibition of second messengers, notably phosphatidylinositol 3′ kinase is inhibited by D-3-deoxy-3-azido-myoinositol and phospholipase C by ether lipids, two of which, ET-18-OME and SRI 62-834, are undergoing clinical evaluation. The author observes that since the biochemical pathways involved in signal transduction are linked to cell proliferation, medicinal chemists will be producing a new generation of antimetabolites complementary to those in present use.

The design of novel antiendocrine drugs is presented by Michael Jarman. New anti-oestrogens are discussed in the context of the facile metabolism of tamoxifen and its retention of partial agonist activity, which may contribute to the emergence of tumour resistance. Several congeners are currently undergoing clinical study, including droloxifene, teremefin, TAT-59 and idoxefine (CB7432). The last compound has a substantially improved affinity for the oestrogen receptor compared with the other compounds. Also in clinical study is the 7-substituted oestradiol derivative, ICI 182,780 which is devoid of agonist activity. Blockade of oestrogen biosynthesis can be achieved with aminoglutethimide, though the side-effects of this drug (sedation and corticosteroid depletion) have prompted the search for an alternative. Several compounds of varying potency are undergoing clinical study, including rogletimide, CGS 16949A and CGS 20267. Amongst the steroidal inhibitors are 4-hydroxyandrostenedione and MDL 18962. Since ovarian and testicular steroid biosynthesis is under LHRH control following its release from the hypothalamus, LHRH agonists have been used, frequently in combination with an inhibitor of oestrogen or androgen biosynthesis, to ablate hormone levels. The synthesis of new anti-androgens, specifically inhibitors of 17α-hydroxylase/$C_{17,20}$lyase, based upon the model compound ketoconazole, is described. Cyclohexyl-4-pyridyl acetate is a lead compound from a group of 4-pyridylacetic acid esters which exhibit good binding affinities. Another important target for anti-androgen drug design is testosterone 5α-reductase, and modelling and structure–activity studies are described. In his concluding discussion the author observes that, in view of the plethora of highly potent and selective aromatase inhibitors, the value of this target in breast cancer chemotherapy will surely be established in the near future.

Paul Workman writes on the design of novel bioreductive drugs, outlining the biology of hypoxia and its association with both radio- and chemoresistance. The nitroimidazole radiosensitisers are elaborated as a major strategy to circumvent radioresistance. The clinical limitations of metronidazole (potency) and misonidazole (neurotoxicity) led to the development of etanidazole and pimanidazole and subsequently of the more promising 'mixed function' compounds containing both the sensitising nitro group and an aziridine function, e.g. RSU 1069 and RB 6145. The heightened cytotoxicity of nitroimidazoles in hypoxic, as compared with oxic, cells has led to the development of a class of cytotoxic chemicals which is activated by reduction in hypoxic environments. In addition to nitroimidazoles, other classical examples are mitomycin C and the indoloquinone E09. More recently the benzotriazine di-*N*-oxides, notably SR4233, have been discovered. The latter is unique in forming strand breaks rather than DNA adducts post reduction. The one- and two-electron reducing requirements for the various activations and the associated enzymology is discussed, as is the possibility of profiling these drugs to the bioreductive phenotype of tumours. The overall approach is presented as exploiting a common established defect of solid tumours.

The topic of 'therapeutic drug monitoring and dose optimisation in oncology' is presented by Merrill Egorin. His theme is the need to achieve optimal dosing of anti-cancer drugs, which invariably possess low therapeutic indices, in individual patients. A sound background to pharmacokinetic and pharmacodynamic monitoring is provided, together with a critique of three dosing strategies that could be adopted. These are 'empiric dosing' (e.g. $mg\,m^{-2}$ as recommended for phase II studies); 'adaptive dosing' (in which patient, disease and drug characteristics are assessed in a regression model to provide an individual dose recommendation against a population database); and 'adaptive control with feedback dosing' (which allows more closely for inter-patient pharmacokinetic variability through refinements of the pharmacokinetic model on an individual basis). Approaches to the analysis of population pharmacokinetics and limited sampling strategies are clearly presented and analysed. The need for appropriate resources is stressed, as is the requirement for sensible cost–value benefit of such investigations. The author also points to the need to initiate population pharmacokinetic/pharmacodynamic modelling at the earliest stages of the clinical evaluation of a new drug, since the basic information required to undertake individual dose optimisation will be difficult, if not impossible, to obtain once the drug is established.

Gilberto Schwartsmann reviews current strategies in anticancer drug discovery within the EORTC. The rate of discovery of clinically useful drugs is addressed briefly and the point made that the major impact has been in the treatment of relatively rare cancers (leukaemias, lymphomas, paediatric tumours). This is attributed to the predominance of transplantable mouse leukaemias, either as prescreens or as major screening vehicles, in the tumour panels adopted for assessment and quantification of preclinical anti-tumour properties.

It is also no doubt a consequence of the drug discovery philosophy which has relied on large-throughput random screening, rather than rational drug design. The inception and evolution of the NCI compound-oriented screening programme and its benefits and shortcomings are reviewed. Also outlined is the current NCI screening programme, which is disease oriented and embraces calibrated panels of human tumour cell lines of given tissue types. The organisation of the EORTC drug discovery initiative is discussed, indicating the role played by the New Drug Development Office in coordinating and integrating the efforts of the University of Freiburg and the several collaborative EORTC preclinical groups. The chapter concludes with a brief commentary on the EORTC contributions to the development of several promising new agents including taxol, taxotere, topotecan, CPT-11, the anthrapyrazoles and suramin.

K.R. Harrap